# Subliminally and Supraliminally Acquired Long-Term Memories Jointly Bias Delayed Decisions

**DOI:** 10.3389/fpsyg.2017.01542

**Published:** 2017-09-12

**Authors:** Simon Ruch, Elizabeth Herbert, Katharina Henke

**Affiliations:** ^1^Department of Psychology, University of Bern Bern, Switzerland; ^2^Center for Cognition, Learning and Memory, University of Bern Bern, Switzerland; ^3^School of Physiology, Pharmacology and Neuroscience, University of Bristol Bristol, United Kingdom

**Keywords:** subliminal stimulation, decision-making, unconscious processing, long-term memory, relational learning, hippocampus

## Abstract

Common wisdom and scientific evidence suggest that good decisions require conscious deliberation. But growing evidence demonstrates that not only conscious but also unconscious thoughts influence decision-making. Here, we hypothesize that both consciously and unconsciously acquired memories guide decisions. Our experiment measured the influence of subliminally and supraliminally presented information on delayed (30–40 min) decision-making. Participants were presented with subliminal pairs of faces and written occupations for unconscious encoding. Following a delay of 20 min, participants consciously (re-)encoded the same faces now presented supraliminally along with either the same written occupations, occupations congruous to the subliminally presented occupations (same wage-category), or incongruous occupations (opposite wage-category). To measure decision-making, participants viewed the same faces again (with occupations absent) and decided on the putative income of each person: low, low-average, high-average, or high. Participants were encouraged to decide spontaneously and intuitively. Hence, the decision task was an implicit or indirect test of relational memory. If conscious thought alone guided decisions (= H_0_), supraliminal information should determine decision outcomes independently of the encoded subliminal information. This was, however, not the case. Instead, both unconsciously and consciously encoded memories influenced decisions: identical unconscious and conscious memories exerted the strongest bias on income decisions, while both incongruous and congruous (i.e., non-identical) subliminally and supraliminally formed memories canceled each other out leaving no bias on decisions. Importantly, the increased decision bias following the formation of identical unconscious and conscious memories and the reduced decision bias following to the formation of non-identical memories were determined relative to a control condition, where conscious memory formation alone could influence decisions. In view of the much weaker representational strength of subliminally vs. supraliminally formed memories, their long-lasting impact on decision-making is noteworthy.

## Introduction

Decision-making is the cognitive process of collectively integrating relevant knowledge to determine the optimal option among several possibilities. Decision-making is considered to depend on conscious deliberation (Simon, 1955; Bettman et al., 1998; Kahneman, 2003; Newell and Shanks, 2014) because good decisions require long-term episodic memory, a proper analysis and integration of information, strategic reasoning and formal logic. Increasing evidence suggests that decision-making is also influenced by unconscious mental processes (Betsch and Gloeckner, 2010; Custers and Aarts, 2010; Baumeister et al., 2011; van Gaal et al., 2012; Creswell et al., 2013; Hassin, 2013). Unconscious cognition might be most relevant in situations where decisions have to be made intuitively, for example if a deliberate analysis of decision alternatives and their possible consequences is not possible (Betsch and Gloeckner, 2010; Zander et al., 2016). Here, we ask if intuitive decisions benefit from unconscious knowledge, i.e., from memories that were formed outside of awareness. We further ask how such unconscious knowledge interacts with consciously formed memories during decision-making. Are decisions and choices exclusively determined by conscious information, or do humans integrate all available conscious and unconscious knowledge while making decisions? To address these questions, we tested whether subliminal information, i.e., information presented below the threshold of awareness, is stored in memory to guide later decisions unconsciously. We further explored whether such subliminally acquired unconscious knowledge would interact with later encoded supraliminal information in guiding decision-making.

Prominent evidence for unconscious influences on decision processes is provided by priming studies, in which subliminal stimuli are presented as primes to alter the conscious processing of subsequently presented target stimuli (for reviews see e.g., Van den Bussche et al., 2009; Ansorge et al., 2014; Henson et al., 2014). Subliminal primes were found to facilitate responses to subsequently presented suprathreshold targets—either by reducing response latency or by increasing response accuracy—if primes and targets are associated with the same response. Importantly, priming has been observed even if primes were perceptually different from targets but shared the same semantic category, and even if prime stimuli were never used as targets and thus were not directly associated with a specific response (Ortells et al., 2016). These findings suggest that subliminally presented information can influence decisions not only by triggering pre-established motor responses, but by activating conceptual knowledge about the upcoming target (Van den Bussche et al., 2009). In addition to facilitating responses to target stimuli, subliminal primes were found to bias “free choices” in target-absent trials where participants could freely choose between response alternatives (Schlaghecken and Eimer, 2004; Klapp and Haas, 2005; Kiesel et al., 2006; Milyavsky et al., 2012; Parkinson and Haggard, 2014; Ocampo, 2015, 2016). Hence, the processing of subliminal stimuli may not only influence the speed and accuracy of responses concerning a supraliminal target but may bring forth new behaviors in situations where choices depend entirely on subjective preferences.

Subliminal priming is notoriously short-lived suggesting that unconsciously processed information might not be stored in long-term memory and thus might fail to inform delayed decisions. The influence of subliminal primes usually dissipates within a few hundred milliseconds and is restricted to the immediately following target (Forster et al., 1990; Ferrand, 1996; Greenwald et al., 1996; Mattler, 2005; Dupoux et al., 2008). Although, the precise mechanisms of subliminal priming are still debated (Henson, 2003; Kiesel et al., 2007; Kouider et al., 2007; Gomez et al., 2013; Henson et al., 2014), there is consensus that subliminal primes briefly enhance the accessibility of pre-established perceptual or semantic knowledge or stimulus-response associations. This enhanced accessibility facilitates the conscious processing of perceptually or semantically prime-related targets but does not leave an enduring memory trace (but see Bodner and Masson, 2001, 2014 for an alternative view).

Besides the evidence for a rapid decay of subliminal priming there is a growing literature reporting long-term effects of subliminal stimuli, suggesting mechanisms of unconscious encoding that yield long-term memory traces. The repeated exposure to the same subliminal stimulus was found to enhance perceptual sensitivity to the same stimulus‘ features when presented delayed and supraliminally, thereby facilitating conscious stimulus identification and classification (Seitz and Watanabe, 2009; Seitz et al., 2009; Rosenthal and Humphreys, 2010; Atas et al., 2013; Pascucci et al., 2015). Hence, subliminal perceptual learning can durably enhance the saliency of a stimulus and might thus boost its impact on delayed decisions. Several studies revealed that humans can even learn entirely novel associations between subliminal stimuli and supraliminal events or between subliminal cues and specific behavioral outcomes. In classical conditioning experiments, initially neutral subliminal stimuli began to elicit arousal-related physiological responses if these stimuli were repeatedly paired with an arousing event such as an electric shock (Wong et al., 2004; Both et al., 2008; Raio et al., 2012; Lipp et al., 2014; Jensen et al., 2015). Operant conditioning experiments revealed that subliminally presented stimuli helped to improve task performance if these stimuli reliably predicted which behavior would lead to success in the next trial (Pessiglione et al., 2008; Palminteri et al., 2009; Pine et al., 2014; Mastropasqua and Turatto, 2015). Similar results were obtained if initially irrelevant subliminal stimuli were repeatedly followed by a switch of tasks in task-switching experiments or were paired with specific target categories in priming experiments: with enough practice, participants improved on switch trials when subliminal cues had predicted the task switches (Zhou and Davis, 2012; Manly et al., 2014; Farooqui and Manly, 2015) and were faster at classifying targets that were predicted by a preceding subliminal prime (Custers and Aarts, 2011; Marcos Malmierca, 2015). Finally, dichoptic stimulation experiments showed that the repeated presentation of a sequence of subliminal events allowed participants to later identify this same sequence despite their inability to detect the underlying events (Rosenthal et al., 2010, 2016). These findings demonstrate that humans can learn about the predictive value of subliminal stimuli and can utilize this knowledge to improve their decisions. However, these findings also suggest that subliminal learning might be slow and might only yield rigid knowledge that cannot be transferred flexibly to other tasks or contexts (Rosenthal and Soto, 2016). Indeed, subliminal learning in these studies required extensive training and manifested only as improvement within the very task that participants had been trained on. Unconsciously acquired knowledge might thus be too weak to interact with consciously formed memories during decision-making.

Studies investigating the role of consciousness in memory yielded evidence that humans are able to rapidly form novel relational memories between subliminal stimuli and may flexibly retrieve these unconscious relational memories in new contexts when facing various decision tasks (e.g., Reber and Henke, 2011; Chong et al., 2014; Reber et al., 2014; Kawakami and Yoshida, 2015). For example, the subliminal presentation of faces paired with written occupations later biased participants' conscious decisions about the income of these individuals if the faces were re-presented supraliminally for conscious inspection. Faces that had been paired with a high-wage occupation during subliminal stimulation later received higher income ratings than faces paired with a low-wage occupation (Ruch et al., 2016). This effect was observed despite the fact that participants had not noticed the subliminal stimuli, did not consciously remember the faces and occupations, and rated the income based on their mere intuition. Similar effects of subliminally presented face-occupation pairs on decision-making were obtained if participants were later asked about the education, regularity of income, or creativity of the work performed by the depicted individuals (Duss et al., 2011). The subliminal impact on decisions was observed after delays of up to 25 min and was achieved with only one subliminal encoding trial (Duss et al., 2011; Ruch et al., 2016). These findings evince rapid binding of subliminal stimulus pairs into relational long-term memories that can be used flexibly in novel tasks and contexts. One-trial encoding, relation formation, and long-term representation of relations in a flexible format are the defining properties of episodic memory (Cohen and Eichenbaum, 1993; O'Reilly and Rudy, 2001; Henke, 2010). Because memories formed from subliminal stimuli lived up to this computational definition (Reber et al., 2012; Duss et al., 2014; Ruch et al., 2016) it must be assumed that episodic memory formation may proceed with and without conscious awareness (Henke, 2010; Dew and Cabeza, 2011; Hannula and Greene, 2012; Olsen et al., 2012; Ortu and Vaidya, 2013). Such a notion is all the more surprising as the formation and retrieval of episodic memory, mediated by the hippocampus, was long thought to depend on conscious awareness (e.g., Moscovitch, 1995; Squire and Zola, 1996; Tulving, 2002). This view was inspired by the finding that hippocampal lesions impaired declarative (conscious) memory for facts and episodes but spared non-declarative (non-conscious) forms of memory such as motor skill learning, priming, and conditioning (Squire and Zola, 1996). Studies using implicit or indirect tests to examine non-conscious forms of episodic memory (e.g., Rose et al., 2002; Henke et al., 2003; Greene et al., 2007; Hannula and Ranganath, 2009; Duss et al., 2014; Ryals et al., 2015; Rosenthal et al., 2016) revealed, however, that the function of the hippocampus does not depend on consciousness but on the type of processing involved at learning: the hippocampus mediates the rapid relational encoding and storage of both consciously and unconsciously apprehended information (for reviews see Henke, 2010; Dew and Cabeza, 2011; Hannula and Greene, 2012; Olsen et al., 2012; Ortu and Vaidya, 2013). Both conscious and unconscious relational memories were stored for long-term (Ruch et al., 2016) within the hippocampal memory space (Züst et al., 2015) with overlapping conscious and unconscious relational memories interacting with each other (Henke et al., 2013; Züst et al., 2015).

**Figure 1:**
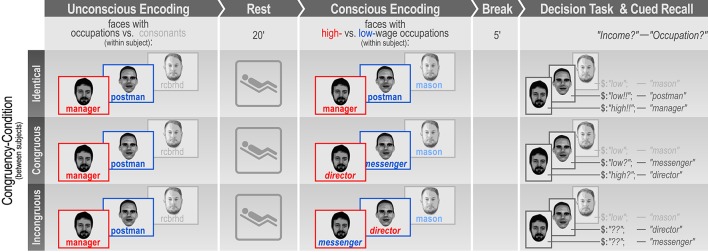
Experimental Design. Participants were presented with subliminal combinations of faces and written high- (blue) or low-wage (red) occupations or consonant strings (light gray: control condition). Subliminal encoding was followed by a rest period of 20 min. Next, participants consciously encoded the same faces presented supraliminally now combined with either the same occupations, congruous or incongruous occupations. The decision task followed after another break. All faces were shown again, without any occupations, for participants to rate each person's putative income and then to try to recall the person's occupation as learned before. The individuals depicted in this figure gave written informed consent to publication of their image.

Here we ask whether unconsciously and consciously acquired relational memories formed in one encoding trial would interact to guide delayed decision-making. What happens if unconscious and conscious memories contain conflicting contents that exert an opposing influence on decisions? How are the two conflicting strands of information weighted? In a day-to-day scenario, this type of conflict may occur if, for example, one reads a news article, which portrays a particular politician in a negative light after having unconsciously integrated surrounding information presenting the same politician in a positive light. How much influence would the unconsciously acquired information have on one's voting behavior? To study potential interactions between unconsciously and consciously formed memories during decision-making, we presented participants with subliminal pairs of faces and written occupations or faces and consonant strings (control condition) for unconscious encoding. Following a resting period of 20 min duration, participants consciously (re-) encoded the same faces presented supraliminally along with either the same written occupations, occupations congruous to the subliminally presented occupations (same wage-category), or incongruous occupations (opposite wage-category). Faces unconsciously encoded with consonant strings (control condition) were presented along with new occupations. Five minutes following the conscious acquisition of face-occupation associations, we assessed the influence of unconsciously and consciously formed memories on decision-making. Participants were presented with the same faces (no occupations) and were instructed to rate each person's putative income on a four-point scale: low, low-average, high-average, or high income. Importantly, participants were encouraged to respond spontaneously and intuitively when deciding about the income of a person. The decision-making task thus constituted an indirect (implicit) test of memory for subliminally (unconsciously) and supraliminally (consciously) presented face-occupation pairs. If conscious thought alone guided income decisions, supraliminal information should determine decision outcomes independently of its congruency with subliminal information. We hypothesized, however, that subliminally formed memories would interact with consciously formed memories to exert an influence on decision-making. To test this hypothesis, we measured the bias that consciously encoded face-occupation pairs exerted on income decisions and examined how this bias was modulated by subliminally presented occupations that were identical, congruous, or incongruous to the supraliminally presented occupations. We expected the decision bias provoked by supraliminal occupations to be increased, reduced, or unchanged depending on whether the same/congruous occupations, incongruous occupations, or no occupations (control condition) had been presented subliminally. Faces that were supraliminally shown as high-earning “manager” or low-earning “postman” should thus yield higher or lower income ratings, respectively, if they were subliminally encoded with the same or a congruous occupation instead of an occupation of the opposite wage-category (see Figure 1).

## Materials and methods

### General procedure

We presented subliminal faces paired with written high- and low-wage occupations (experimental condition) and faces paired with consonant strings (control condition) for unconscious relational encoding. Participants were ignorant of subliminal presentations. They performed an attention task that directed their focal attention to the screen center while subliminal faces and occupations were presented around the screen center (the method of Degonda et al., 2005). This task ensured that subliminal stimuli were attended, which is important to engage unconscious processing of subliminal information (Marzouki et al., 2007; Finkbeiner and Palermo, 2009). Unconscious encoding was followed by a delay period of 20 min during which participants were asked to relax. Quiet resting is known to improve consolidation and retention of newly acquired memories (Brokaw et al., 2016) and thus was expected to enhance unconscious influence on subsequent decisions. Following resting, participants encoded the same faces presented supraliminally. Faces that had been unconsciously encoded with high- and low-wage occupations were supraliminally presented along with either the same high- or low-wage occupations, with occupations congruous to the subliminally presented occupations (same wage-category), or incongruous occupations (opposite wage-category). Control faces that had been flashed subliminally with consonant strings were supraliminally presented along with new high- or low-wage occupations. These faces served as baseline condition in which conscious information alone could bias subsequent decision-making. While all participants encoded control faces, the three congruency conditions were implemented in different subgroups (between-subjects factor). This was necessary to reduce memory load and because the German language does not provide enough distinct occupation words to implement every condition in each participant. Participants encoded the supraliminally presented material with an incidental learning task. They were not informed of a later recall test.

Conscious encoding was followed by a brief delay during which participants again were asked to relax (2 min) and then were instructed about the decision task (~3 min). To measure the common influence of unconsciously and consciously encoded information on decision-making, participants were again presented with all faces to decide on the income of each person (low, low average, high average, high). They were encouraged to respond spontaneously and intuitively and to listen to their gut feeling when deciding on the income. Following each income decision, participants were also asked to remember the actual occupation of the person.

Once the decision task and recall test were completed, participants were questioned about the strategies they had used to rate the income of the depicted persons (open-ended question). This interview should reveal the actually employed strategies in the income rating task. Next, participants were queried on their expectancy and subjective awareness of masked stimuli during the experiment. This inquiry was succeeded by objective awareness tests in which masked stimulus presentations were immediately followed by forced-choice questions to test explicit stimulus visibility.

The sequence of tasks and delay periods was designed to provide optimal conditions for studying the joint impact of unconsciously and consciously formed long-term memories on decision-making. The delay periods that followed the two encoding runs allowed us to demonstrate that both unconscious and conscious influences on decision-making depended on long-term memory. The duration of delays was chosen such that ~30 min passed between unconscious encoding and the decision task. This experiment replicates and extends a previous experiment (Ruch et al., 2016), which suggested that unconsciously formed relational memories can bias decision processes across delays of up to half an hour. Importantly, unconscious encoding preceded conscious encoding by a delay period of 20 min. This guaranteed that subliminal stimuli had to be stored for long-term in order to shape subsequent behavior.

### Participants

A total of 96 right-handed volunteers participated in this study. Sample size was determined based on previous studies (Degonda et al., 2005; Duss et al., 2011; Ruch et al., 2016) and by the number of participants needed to fully counterbalance stimulus lists across conditions. Three participants failed to adhere to instructions, one was under the influence of psychiatric medication, and technical problems occurred during the testing of three further participants. Data of these seven participants were thus excluded from analysis. The remaining sample consisted of 69 women and 20 men between 19 and 59 years of age (mean = 28.15 years, *SD* = 11.10). Participants were assigned to 3 groups that corresponded to the three experimental conditions “identical,” “congruous,” “incongruous”: identical *N* = 32 (same face-occupation pairs given for subliminal and supraliminal encoding), congruous *N* = 29 (same faces plus semantically congruous occupations), and incongruous *N* = 28 (same faces plus semantically incongruous occupations). Experimenters were aware of the group assignment. We used a cover story (“we study attention and person perception”) to keep participants naïve regarding subliminal presentations and the study rationale. This was necessary to render participants susceptible to subliminal information (Verwijmeren et al., 2013). We obtained written semi-informed consent before experimentation. At the end of the experiment, participants were fully debriefed about the study purpose and the presence of subliminal messages and were reminded of the possibility to withdraw their consent to the use of their data. The study was carried out in accordance with the Helsinki declaration and the recommendations of the local ethics committee “Kantonale Ethik Kommission (KEK) Bern, Switzerland.” The study protocol and the consent procedure were approved by the local ethics committee.

### Tasks

#### Unconscious encoding

**Figure 2:**
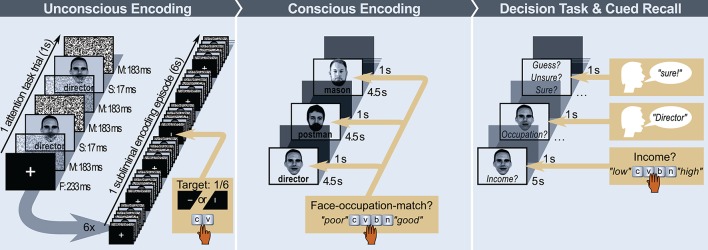
Schematic illustration of experimental tasks. Unconscious Encoding: One subliminal encoding episode in which one subliminal stimulus (S: face with occupation word) is presented 12 times in between masks (M). One encoding episode consists of 6 attention task trials. In one of these trials, the fixation screen (F) contains a target stimulus (“−” or “|” instead of “+”); Conscious Encoding: Sequence of three supraliminal encoding trials. Participants indicated by button press how well the indicated occupation matched a person ([C]: “poor,” [V]: “poor-average,” [B]: “good-average,” or [V]: “good” match); Decision Task and Cued Recall: Participants first decided on the suspected income of the person ([C]: “low,” [V]: “low-average,” [B]: “high-average,” or [V]: “high” income), then tried to remember and say the occupation associated to this face, and finally indicated whether they randomly guessed, had a hunch but were unsure, or certainly remembered the occupation; The individuals depicted in this figure gave written informed consent to publication of their image.

We used an established forward and backward masking paradigm (Duss et al., 2011, 2014; Ruch et al., 2016) to render face-occupation pairs subliminal for unconscious encoding. Subliminal stimuli were embedded in an attention task. The attention task required participants to hit a key each time a regularly occurring fixation screen (F) was replaced by a screen displaying a horizontal (F_H_) or vertical (F_V_) line segment instead of a complete fixation cross (F_+_). Each trial of the attention task took 1 s and started with a fixation screen (F_+_, F_H_, or F_V_) that was followed by four visual noise masks (M) and two subliminal presentations (S) constituting the sequence F_+/H/V_–M–S–M–M–S–M. Fixation screens were presented for 233 ms, subliminal stimuli for 17 ms, and masks for 183 ms. Each face-occupation pair was presented 12 times in sequence during six adjacent attention trials spanning 6 s. During this time, one in six fixation screens featured a target (F_H_ or F_V_) that participants had to respond to (see Figure 2A and Supplementary Videos 1, 2 for an illustration of one subliminal encoding episode). The added total exposure time of a subliminal face-occupation pair was 204 ms (12^*^17 ms). Each participant encoded 40 subliminal faces, whereof 10 faces were combined with a low-wage occupation, another 10 faces with a high-wage occupation and the remaining 20 faces with a non-sense consonant string. Unconscious encoding took 4 min.

#### Resting phase

Following unconscious encoding, participants relaxed on a comfortable reclining seat for 20 min to allow for a period of undisturbed memory consolidation. Half of participants in each experimental condition (identical, congruous, incongruous) relaxed fully and half were required to perform an undemanding acoustic vigilance task, namely to hit a button when hearing a tone played at random intervals of 15–25 s. This manipulation served the aim of finding out whether a deeper relaxation (no task vs. a vigilance task) would benefit memory consolidation. Self-reports in the no-task condition and the number of missed target tones in the vigilance task condition revealed that participants of both groups were equally inclined to fall asleep, suggesting that both conditions were very relaxing. The factor resting condition (no task/vigilance task) was therefore excluded from analyses but the pattern of findings remains the same if it is included.

#### Conscious encoding

Following rest, participants were presented with supraliminal displays of the same 40 faces that had been flashed subliminally (Figure 2). Depending on the experimental condition (varied between subjects), faces were combined with either the same written occupations, congruous occupations or incongruous occupations. Ten of the 20 control faces that had been combined with letter strings for subliminal presentations were now presented with a high-wage occupation and 10 with a low-wage occupation (control condition). Each face-occupation pair was presented for 4.5 s. The incidental encoding task required participants to give a subjective, intuitive response of how well an occupation matched a person (poor match, poor-average match, good-average match, good match).

#### Decision task and explicit memory test

We assessed the influence of unconsciously and consciously acquired face-occupation pairs on income ratings regarding these same persons (Figure 2). The previously presented 40 faces were displayed again, each for 5 s and without written occupations, for conscious inspection. Participants were asked to decide on the suspected income of each person on a scale ranging from 1 to 4: low (1), low-average (2), high-average (3), high (4; response by key press). They were encouraged to respond spontaneously and intuitively and to focus on the depicted person (rather than on the previously presented supraliminal occupation). The intuitive strategy and the focus on the depicted person should allow the penetrance of the influence of subliminally presented, unconsciously acquired information on decision-making. We used a rating scale rather than a binary response format because we were interested in measuring the average influence of both subliminally and supraliminally encoded occupations on decision-making (occupations can conflict in terms of the associated wage, yielding in combination an average wage) and because the presented occupations were associated with an either high or low wage but not extreme endpoints of wages. Scales allow for more variance and are therefore better suited for the measurement of implicit, unconscious influences. Furthermore, the persons whose income should be rated were selected to look neither wealthy nor poor. Hence, without any knowledge of a person's occupation participants would be inclined to select a mid-range income. Following each income decision, participants were required to try to remember and say the occupation they had earlier read below the face of the person. They were asked to indicate whether they randomly guessed the occupation (“guess”), had a hunch but felt uncertain about their answer (“unsure”), or felt sure they remembered the occupation (“sure”). This cued recall test served to determine the quality of conscious episodic memory.

#### Assessment of decision-making strategy

Following the experiment, participants were asked whether they had used a specific strategy to decide on the income (open-ended question). This interview should reveal the actually employed strategies in the income rating task.

#### Awareness test

At the end of the experiment, we determined subjective awareness of subliminal stimuli. Participants were asked about any potential awareness of the study purpose and the presence of subliminal stimuli with a series of increasingly specific questions. Finally, we measured awareness of subliminal faces and subliminal occupation words using two objective forced-choice awareness tests (as described in Ruch et al., 2016, experiment 1). We implemented 20 trials for each test to achieve equal test strength as in the experiment where 20 trials constituted an experimental condition. A new set of 20 face-occupation combinations was used for subliminal encoding in both the face-awareness test and the occupation awareness test. In both awareness tests, a trial consisted of the subliminal presentation of a face-occupation combination over 6 s (as in the experiment) immediately followed by either a forced-choice between two supraliminally shown faces (target face and a foil face) or between the category “high-wage occupation” and “low-wage occupation.” If the chosen wage-category corresponded to the written occupation flashed with a face, the answer was scored as correct. Subliminal stimuli were presented using the same masking paradigm and attention task as in the experiment. Instructions required participants to try to consciously discern the subliminally flashed faces and words, or fragments thereof, in order to inform choices. The order of the two awareness tests was counterbalanced between participants. The percentage of correctly identified faces and wage categories was the dependent variable.

### Materials

We used 80 grayscale images of male faces from the FERET database (Phillips et al., 1998, 2000) which received average income ratings by students in a pilot study that we conducted in preparation of this study. Of the 80 faces, 40 were used in the experiment (20 in the experimental and 20 in the control condition) and 40 in the two awareness tests (20 targets and 20 foils in the forced-choice test for face awareness). Luminance and contrast of all images were equalized. Faces were assigned to 8 lists of 10 faces arranging for an equal distribution of mean income ratings (based on the pilot data), emotional facial expressions, facial hair, and age across lists. Across participants, each face was presented an equal number of times in the experiment and the awareness tests. Within the experiment, each face was presented an equal number of times with a high- and a low-wage occupation and with a consonant string (control condition) for subliminal encoding and with an identical, a congruous and an incongruous occupation for conscious encoding.

We came up with 40 pairs of semantically congruous occupations—such as “director” and “manager” or “postman” and “messenger.” Twenty pairs corresponded to common high-wage occupations (e.g., director and manager) and 20 pairs to common low-wage occupations (e.g., postman and messenger). The typical wage of occupations was assessed in a pilot study (*N* = 34) in which participants estimated the income each occupation would yield on a 5-point scale ranging from −2 (low) to +2 (high). The estimated income of high-wage occupations ranged from 0.17 to 1.60 (scale from −2 to+2) with a mean of 0.88 (*SD* = 0.07). The income of low-wage occupations ranged from −0.42 to −1.61 with a mean of −0.92 (*SD* = 0.05). Assuming that average income ratings on a 5-point scale are normally distributed, we estimated that high-wage occupation words reflected the upper 41% and low-wage occupations the lower 34% of all possible wages. The total of 80 occupation words were assigned to 4 lists of 10 high-wage and 10 low-wage occupations, of which two lists contained congruous occupations required for the congruous condition in the experiment (A and A′ and B and B′). In the incongruous condition, high- and low-wage occupations were swapped between faces from subliminal to supraliminal encoding to induce semantic conflict. A and B lists were used in the experiment and awareness tests, respectively, in a counterbalanced fashion. Words in the four lists were matched regarding mean logarithmic word frequency (retrieved from the Leipzig Corpora Collection, http://corpora.uni-leipzig.de/) and character count. The 20 consonant strings required for the subliminal presentations in the control condition were built by randomly assembling the consonants contained in the selected occupation words. Consonant strings corresponded to occupation words regarding their character count. Supplementary Table 1 provides a list of all occupation words, with word frequency, character count, and estimated average income (as rated by an independent sample of 34 participants). Supplementary Table 2 provides a list of all consonant strings.

Stimuli were presented using the software Presentation® (Version 16.2, Neurobehavioral Systems, http://www.neurobs.com) on a standard Dell Notebook running Windows XP. A Digital Light Processing (DLP) projector BenQ SP831 projected the face-occupation pairs (occupation written below face) onto a screen in front of participants in a visual angle of 15° width with a resolution of 1,024 × 768 at a refresh rate of 60 Hz.

Responses were recorded with a standard USB keyboard. Participants responded using their right hand, index to little finger, tapping the four neighboring keys [c], [v], [b], [n].

## Results

### Data analysis

The dependent variable in the decision task was the subjects' mean income rating (four possible answers: 1 = low, 2 = low-average, 3 = high-average, 4 = high) for the 10 faces of each of the four within-subject conditions, i.e., for faces consciously encoded with high- vs. low-wage occupations and unconsciously encoded with occupations vs. consonant strings. The dependent variable in the cued recall test was the percentage of correctly recalled occupations for each condition. Each dependent variable was used in a 2 × 2 × 3 repeated measures ANOVA with the two within-subject factors *Occupation/Consonant* (subliminal occupation word vs. consonant string) and *High-/low-wage* (referring to occupations presented supraliminally for conscious encoding) and the between-subjects factor *Congruency* (identical, congruous, and incongruous).

We assessed normality of the two dependent variables separately for each of the four within-subject conditions, i.e., for faces consciously encoded with high- vs. low-wage occupations and unconsciously encoded with occupations vs. consonant strings. Although, Kolmogorov–Smirnov tests suggested that mean income ratings deviated from normality [all *D*_(89)_ > 0.09, all *p* < 0.05], histograms, Q-Q plots, and skewness (range: [−1, 1]) and kurtosis (range: [−2, 2]) values indicated a normal distribution. We therefore used parametric tests to examine our hypotheses. Note, however, that the observed pattern of results (see Section Decision-Making) was replicated when we standardized the raw income ratings per participant (z-transformation) to obtain mean ratings that satisfied all criteria of normality [Kolmogorov–Smirnov tests: all *D*_(89)_ < 0.08, all *p* > 0.20, range of skewness & kurtosis: [−1, 1]]. The pattern of results was also replicated when using non-parametric repeated measures ANOVAs as implemented in the function *ezPerm* of the *R*-package “*ez*” (Lawrence, 2016). Percentages of recalled occupations were not normally distributed due to the participants' low recall performance (see Section Conscious Retrieval). Non-normality was suggested by histograms, Q-Q plots, and highly significant Kolmogorov–Smirnov tests [all *D*_(89)_ > 0.21, all *p* < 0.001]. The low overall recall performance prevented a meaningful analysis and interpretation of the influence of subliminal information on the conscious recall of supraliminal information.

### Attention task/unconscious encoding

Mean hit rate in the attention task was 88.20% (*SD* = 9.65) which indicates that participants closely attended to the center of the images provided for the attention task and for subliminal encoding.

### Conscious retrieval

Participants recalled the occupations of 10.09% of the supraliminally displayed face-occupation pairs (*SD* = 7.35%). This low recall performance (10% means 4 of the 40 learned face-occupation pairs) probably owes to the incidental encoding instruction, the large amount of learning material, and the fact that only one encoding run was provided. Recall performance was neither affected by wage (factor *High-/low-wage)* nor *Congruency* (identical, congruous, incongruous) as suggested by the non-significance of all main effects and all interaction terms in the 2 × 2 × 3 ANOVA (all *p* > 0.134).

### Decision-making

Decision-making was significantly influenced by both the supraliminal and the subliminal face-occupation pairs. Income decisions were strongly biased by the wage associated with supraliminal occupations [main effect of *High-/low-wage*: *F*_(1, 86)_ = 13.187, *p* < 0.001, $\eta_{\text{G}}^{2}$ = 0.033]: participants gave significantly higher income ratings to faces that were supraliminally encoded with high- vs. low-wage occupations. Importantly, this bias was moderated by subliminal occupations as suggested by the significant three-way interaction between *Occupation/consonant, High-/low-wage*, and *Congruency: F*_(2, 86)_ = 3.529, *p* = 0.034, $\eta_{\text{G}}^{2}$ = 0.012. Hence, subliminal and supraliminal information both contributed to participants' income decisions. No other main effect or interaction term reached significance (all *p* > 0.200) in this ANOVA.

**Figure 3:**
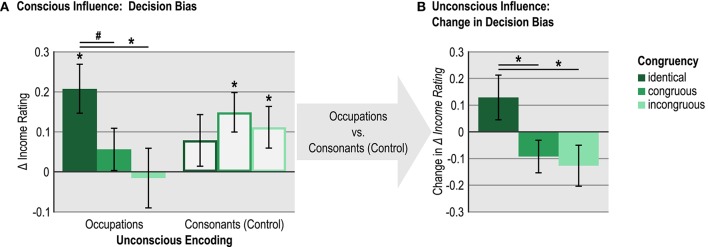
Influences of unconscious and conscious encoding on decision-making. **(A)** Plotted is the difference in income ratings for faces consciously encoded with high- vs. low-wage occupations (i.e., [high]-[low]). This difference reflects the decision bias toward consciously encoded occupations. Results are shown for the between-subjects manipulated experimental condition (identical, congruous, incongruous) and the within-subject manipulated unconscious encoding condition (faces with occupations vs. consonants). The figure illustrates a strong decision bias following unconscious and conscious encoding of identical information, and a reduced or absent bias following encoding of congruous or incongruous unconscious and conscious information. **(B)** Plotted is the change in the bias toward consciously encoded occupations ([high]-[low]) for faces that were unconsciously encoded with occupations instead of consonant strings (i.e., [bias for faces encoded with occupations]-[bias for faces encoded with consonant-strings]). This change reflects the unconscious influence on decision bias: positive scores indicate increased, negative scores decreased decision bias toward the consciously seen occupations due to subliminal encoding of face-occupation pairs. The figure illustrates an increased decision bias following unconscious and conscious encoding of identical occupations, and a reduced bias following encoding of non-identical (congruous or incongruous) information. Displayed are group means with SEMs. #*p* < 0.10, ^*^p < 0.05 (uncorrected two-tailed *t*-tests).

To further examine the interaction between subliminal and supraliminal memory formation, we analyzed the differences in mean income ratings between faces supraliminally encoded with high vs. low-wage occupations separately for the experimental and the control condition (faces subliminally presented with occupations vs. consonant strings) and for each congruency group (see Table 1 for an overview of the mean ratings, and Table 2, tests 1–8, and Figure 3A for the difference scores). Large positive difference scores suggest a strong decision bias toward the wage category of supraliminal occupations. We examined the changes in these difference scores between experimental and control faces (Table 2, test 9–12) and across congruency groups (Table 3, tests 1–6), and we contrasted the changes in difference scores between experimental and control faces across all congruency groups (three-way interactions, see Table 3, tests 7–9). We performed two-tailed *t*-tests for all difference scores and contrasts to assess their significance. We further calculated Bays Factors (BF) for these tests to find out whether the data favored the null hypotheses H0 for *t*-tests that failed to reach significance or for tests where we expected H0 to be true. Bayes Factors were estimated using a half-normal prior distribution with a mode of 0 (reflecting H0, i.e., no difference in income ratings or in difference scores) and a standard deviation reflecting the expected effect size under H1 (Dienes, 2008, 2014). Because participants remembered only ~10% of the supraliminally displayed occupations, we assumed that consciously and unconsciously encoded occupations would influence decision-making with similar effect sizes. Two recent experiments investigating long-term effects of subliminal processing on decision-making (Ruch et al., 2016) yielded an average effect size of 0.4 (effect size was calculated by dividing the reported *t*-values obtained for each of the two experiments by the square root of the respective sample size and then computing the average of the two scores). By multiplying this effect size with the pooled standard deviation of the differences in income ratings obtained in the present study (*SD* = 0.33), we arrived at an expected difference in income ratings of 0.13. This value was used as expected effect size given H1 to calculate Bayes Factors.

**Table 1:** Mean income ratings (M) and standard deviations (*SD*) for each congruency group and each condition (faces consciously encoded with high- vs. low-wage occupations and unconsciously encoded with occupations vs. consonant strings).

**Congruency group**	**Occupations**				**Consonants (control)**				***N***
	**Low**		**High**		**Low**		**High**		
	**M**	***SD***	**M**	***SD***	**M**	***SD***	**M**	***SD***	
Identical	2.396	0.228	2.604	0.319	2.526	0.319	2.605	0.275	32
Congruous	2.529	0.217	2.585	0.254	2.495	0.234	2.644	0.248	29
Incongruous	2.600	0.257	2.584	0.359	2.572	0.255	2.683	0.221	28

**Table 2:** Decision bias toward consciously encoded occupations.

**Unconscious encoding**	**Test#**	**Descriptives**		***t*****-test**			**BF**
**Congruency group**		**M (diff)**	**SEM (diff)**	***t***	***df***	***p***	
**OCCUPATIONS**							
Identical	1	0.208	0.061	3.403	31	0.002	97.443
Congruous	2	0.056	0.053	1.070	28	0.294	1.033
Incongruous	3	−0.015	0.075	−0.206	27	0.838	0.436
Congr. and Incongr.	4	0.021	0.045	0.467	56	0.642	0.487
**CONSONANTS (CONTROL)**							
Identical	5	0.079	0.065	1.221	31	0.231	1.398
Congruous	6	0.149	0.049	3.022	28	0.005	38.360
Incongruous	7	0.111	0.052	2.139	27	0.042	5.214
Congr. and Incongr.	8	0.131	0.036	3.667	56	0.001	274.423
**OCCUPATIONS-CONSONANTS**							
Identical	9	0.129	0.084	1.542	31	0.133	2.268
Congruous	10	−0.093	0.061	−1.522	28	0.139	2.009
Incongruous	11	−0.127	0.077	−1.653	27	0.110	2.585
Congr. and Incongr.	12	−0.109	0.048	−2.261	56	0.028	6.474

*Differences between mean income ratings for faces consciously encoded with high- vs. low-wage occupations are indicated. Difference scores reflect decision bias toward the wage category of the consciously encoded occupations. Scores are reported separately for both unconscious encoding conditions (occupations and consonants) and for each congruency group (identical, congruous, incongruous, and congruous and incongruous combined). Decision bias is further contrasted between faces unconsciously encoded with occupations vs. consonants (tests 9–12). These contrasts reflect the unconscious influence on decision bias; Two-tailed t-tests with uncorrected p-values and Bayes Factors are reported. Bayes factors were calculated using a half-normal prior distribution with mode = 0 (reflecting H0) and standard deviation = 0.13 (reflecting the expected effect size under the alternative hypothesis H1) as suggested by Dienes (2014). See text for source of H1*.

**Table 3:** Difference in decision bias between congruency groups.

**Unconscious encoding**	**Test #**	**Descriptives**		***t*****-test**			**BF**
**Group contrasts**		***M* (diff)**	**SEM (diff)**	***t***	***df***	***p***	
**OCCUPATIONS**							
Identical vs. Congr.	1	0.151	0.081	1.860	59	0.068	3.468
Identical vs. Incongr.	2	0.223	0.095	2.337	58	0.023	6.774
Congr. vs. Incongr.	3	0.072	0.091	0.791	55	0.432	1.049
**CONSONANTS (CONTROL)**							
Identical vs. Congr.	4	−0.070	0.083	−0.850	59	0.399	0.330
Identical vs. Incongr.	5	−0.033	0.085	−0.386	58	0.701	0.431
Congr. vs. Incongr.	6	0.038	0.072	0.523	55	0.603	0.728
**OCCUPATIONS-CONSONANTS**							
Identical vs. Congr.	7	0.222	0.105	2.105	59	0.040	4.557
Identical vs. Incongr.	8	0.256	0.115	2.231	58	0.030	5.111
Congr. vs. Incongr.	9	0.034	0.098	0.351	55	0.727	0.764

*Between-group differences in decision bias toward consciously encoded occupations (differences of the difference between income ratings for faces consciously encoded with high vs. low-wage occupations) are reported separately for faces unconsciously encoded with occupations and consonants. Furthermore, the contrast in the decision bias between faces unconsciously encoded with occupations vs. consonant strings is compared between groups; Two-tailed t-tests with uncorrected p-values and Bayes Factors are reported. Bayes factors were calculated using a half-normal prior distribution with mode = 0 (reflecting H0) and with standard deviation = 0.13 (reflecting the expected effect size under the alternative hypothesis H1) as suggested by Dienes (2014). See text for source of H1*.

Mean income ratings revealed that unconscious and conscious encoding of identical occupations had increased the decision bias, whereas encoding of incongruous as well as congruous occupations reduced the decision bias compared to the control condition, in which conscious encoding alone could influence decision outcomes (Figure 3). Most importantly, decisions were strongly biased toward the wage category of supraliminally processed occupations following the unconscious encoding of identical occupations [Figure 3A; Table 2, test 1: *t*_(31)_ = 3.40, *p* = 0.002, BF = 97.44]. The decision bias was marginal or even reversed following the encoding of congruous [Table 2, test 2: *t*_(28)_ = 1.03, *p* = 0.294, BF = 1.03] and incongruous occupations [Table 2, test 3: *t*_(27)_ = −0.21, *p* = 0.838, BF = 0.44], respectively. Although bias in the incongruous group was negative and did not significantly differ from zero, the Bayes factor of 0.44 suggests that the data were only 2:1 in favor of the null hypothesis, indicating merely anecdotal evidence for the absence of any bias. Importantly decision bias was stronger in the identical group compared to the congruous [Table 3, test 1, *t*_(59)_ = 1.86, *p* = 0.068, BF = 3.47] and incongruous group [Table 3, test 2, *t*_(58)_ = 2.34, *p* = 0.023, BF = 6.77]. In the control condition (subliminal presentation of consonant strings), decisions were biased toward consciously encoded occupations (Table 2, tests 5–7, albeit not significantly so for the identical group) and did not significantly differ between congruency groups (Table 3, tests 4–6, all *p* > 0.39, all BF < 0.73). The change in decision bias between faces that had been presented subliminally with occupations vs. consonant strings failed to reach significance for each congruency group (Table 2, tests 9–11). If the data of the congruous and incongruous group were pooled in a *post-hoc* analysis, decision bias was significantly reduced compared to the control condition [Table 2, test 12, *t*_(56)_ = −2.26, *p* = 0.028, BF = 6.47]. Importantly, the change in decision bias between experimental and control stimuli differed significantly between the identical group and both the congruous and the incongruous groups (both *p* < 0.04, both BF > 4.55; Figure 3B and Table 3, tests 7–8).

In short, decision bias was increased following the subliminal and supraliminal processing of identical occupations, but not semantically congruous occupations. Encoding of congruous and incongruous occupations reduced the decision bias compared to the control condition in which faces were subliminally paired with consonant strings. Although, these findings reveal that subliminal stimulation altered subsequent decisions, the pattern of results might suggest that participants did not encode the semantic content of subliminal words and did not form semantically precise relational memories for face-occupation pairs.

#### Assessment of decision-making strategy

Participants were instructed to decide spontaneously and intuitively and to focus on the depicted person (not the occupation they had earlier read below the face) when making their income decision. These instructions should allow the penetrance of the influence of available unconscious, implicit knowledge on decision-making. Note however, that income decisions in all trials were immediately followed by a direct test of explicit memory: participants were required to remember the supraliminally presented occupation. This second task might have motivated participants to first try to retrieve the person's occupation and then to rate the income accordingly. This explicit-memory-informed strategy might reduce the impact of subliminally presented face-occupation pairs on decision-making. We therefore tried to determine the strategies that participants had used to form their decisions.

We performed a qualitative analysis of participants' answers to the post-experimentally posed question of what strategy they had used to rate the income. 70.1% of participants reported having focused on aspects of the depicted person (e.g., charisma or emotional expression, age, cleanliness, facial features such as hairline, similarity with familiar persons, attractiveness). 34.8% of participants explicitly mentioned that they had responded intuitively (note that responses were non-exclusive). Only 12.4% of participants reported that they had tried to remember the associated occupation first only to then deduce the income of the person. The qualitative pattern of results for the decision-making task did not change when these participants were excluded from the analysis: the critical three-way interaction between *Occupation/consonant, High-/low-wage*, and *Congruency* on income decisions remained significant [*F*_(2, 75)_ = 3.224, *p* = 0.045]. This suggests that findings were not affected by the subset of participants who employed a memory-based decision strategy.

To determine participants' decision strategy in a more objective way, we calculated their “decision accuracy,” i.e., the percentage of decisions that corresponded to the wage-category of the supraliminal occupation (% of ratings 3 and 4 for high- and 1 and 2 for low-wage occupations). We determined each participant's accuracy averaged across all faces as well as separately for faces for which occupation retrieval succeeded vs. failed. Classification accuracy averaged across all faces was low (53.0%, SEM = 0.9%) but exceeded chance level of 50% [*t*_(88)_ = 3.26, *p* = 0.002]. Hence, decisions were only weakly influenced by supraliminally seen occupations, which suggests that participants decided intuitively. Importantly, accuracy was much higher for faces for which participants succeeded vs. failed to remember the supraliminally presented occupation [76.6 ± 3.1% vs. 50.6 ± 1.0%; main effect of *Retrieval*: *F*_(1, 81)_ = 59.715, *p* < 0.001]. Accuracy of income ratings (based on supraliminally presented occupations) exceeded chance level only if occupations were remembered [*t*_(83)_ = 8.56, *p* < 0.001. vs. *t*_(88)_ = 0.55, *p* = 0.583]. This suggests that participants relied on the wage-category of supraliminally presented occupations only in trials, where encoding and later explicit retrieval were successful. However, the explicit retrieval may have been delayed to the second task, where it was demanded. This would reduce the influence of supraliminally formed memories at that moment to a preconscious effect that influenced intuitive decisions along with any unconscious effect due to subliminal encoding. The fact that decision accuracy was only 75% even when occupations were remembered speaks in favor of this assumption.

The rather weak influence of supraliminally seen occupation words on income decisions as well as the fact that only a few of these occupations were remembered might suggest that participants employed an intuitive rather than a memory-based decision strategy. Nevertheless, it is still possible that the mere attempt to retrieve the face-associated occupation altered decision-making even if retrieval failed. To assess this possible interaction between the explicit memory test and the decision-making task, we analyzed the correlation between income decisions and the wage-category of subsequently reported but incorrect occupation words at the single-trial level. Only trials in which participants named an incorrect occupation that was not related to the face-associated supraliminal occupation were analyzed. Furthermore, only those occupations were analyzed for which the associated wage-category could be determined, i.e., occupations that were very similar to one of the occupation words used for this study. Income ratings were strongly correlated with the wage-category of occupations reported in the explicit memory test even if participants failed to recall the correct occupation (*r*_S_ = 0.516, *p* < 0.001, *N* = 1,902 trials). This might suggest that participants tried to come up with an occupation from which they could deduce the income of a face instead of deciding intuitively. However, it is equally likely that participants intuitively decided on the income of a face and then tried to find a matching occupation in the memory test. The fact that participants had to decide rapidly (within 5 s) speaks for the latter interpretation.

#### Assessment of potentially confounding factors

Because *Congruency* was manipulated across subjects, it is important to verify that participants' general decision-making style was comparable across groups and that income decisions varied due to subliminal and supraliminal stimulation, not due to a sampling bias. *Congruency* groups neither differed in their overall income-ratings nor in the way they were influenced by supraliminally presented information, i.e., in their general decision bias. This was suggested by the fact that neither the between-subjects effect *Congruency* [*F*_(2, 86)_ = 1.536, *p* = 0.221, $\eta_{\text{G}}^{2}$ = 0.014), nor the interaction of *Congruency* with the wage-category (*High-/low*) of supraliminal occupations [*F*_(2, 86)_ = 1.051, *p* = 0.354, $\eta_{\text{G}}^{2}$ = 0.005] reached significance in the overall ANOVA. As reported earlier, only the three-way interaction *Occupation/consonant, High-/low-wage*, and *Congruency* reached significance. Hence, the content of subliminal stimuli, not group membership *per-se*, moderated the observed differences in decision-making across *Congruency* groups. This finding was confirmed if the same ANOVA was performed for only those faces that were subliminally encoded with consonant strings (control condition): only the main effect of *High-/low-wage* reached significance [*F*_(2, 86)_ = 11.952, *p* < 0.001, $\eta_{\text{G}}^{2}$ = 0.046]. *Congruency* and the interaction between *Congruency* and *High-/Low-wage* again failed to reach significance (all *p* > 0.468). Hence, participants in the different congruency groups gave similar income ratings and showed a similar decision bias for faces that were not associated with a (identical, congruous, or incongruous) subliminal occupation. Only for faces subliminally presented with occupations, did the interaction between *Congruency* and *High-/Low-wage* reach significance [*F*_(2, 86)_ = 3.337, *p* = 0.040, $\eta_{\text{G}}^{2}$ = 0.069]. Hence, the decision bias varied across *Congruency* groups only with respect to subliminally presented occupation words, not due to an overall group bias.

Furthermore, none of the following variables differed significantly between *Congruency* groups: response latencies during decision making [*F*_(2, 86)_ = 0.558, *p* = 0.575], explicit recall performance [*F*_(2, 86)_ = 0.221, *p* = 0.802], age [*F*_(2, 85)_ = 1.180, *p* = 0.312], hit rate on the attention task given during unconscious encoding [*F*_(2, 86)_ = 0.551, *p* = 0.578), gender distribution [$\chi_{\left(2\right)}^{2}$ = 0.784, *p* = 0.676], awareness of subliminal stimuli (see Section Awareness Test), self-reported decision-making strategy [intuition: $\chi_{\left(2\right)}^{2}$ = 4.26, *p* = 0.119; focusing on aspects of faces or persons: $\chi_{\left(2\right)}^{2}$ = 0.107, *p* = 0.948, focusing on associated occupation: $\chi_{\left(2\right)}^{2}$ = 0.205, *p* = 0.903], and decision accuracy (see Section Assessment of Decision-Making Strategy), i.e., the percentage of income decisions that corresponded to the wage-category of supraliminally seen occupations (all *p* > 0.418). This further suggests that participant groups were highly comparable.

### Awareness test

The inquiry into subjective awareness revealed that no participant had suspected subliminal stimuli or noticed faces, words, or fragments thereof between masks. The objective awareness tests corroborated this result. Performance on the objective face and occupation awareness test did not significantly deviate from the chance level of 50% [face awareness test: mean accuracy = 50.17%, 95% CI [47.65, 52.70], *t*_(86)_ = 0.136, *p* = 0.892; occupation awareness test: mean accuracy = 49.11%, 95% CI [47.02, 51.20], *t*_(88)_ = −0.844, *p* = 0.401]. Note that non-significance does not necessarily mean that participants were truly unaware (i.e., that the null hypothesis is true). It is possible that our tests were not sensitive enough to distinguish between the null and alternative hypothesis (Dienes, 2014). To assess if there was substantial evidence for the absence of awareness, we calculated Bayes Factors (BF) for the two *t*-tests. We assumed that awareness for subliminal stimuli would yield a recognition performance of about 5% above chance level. This is the performance achieved in tests that assessed indirect influences of subliminal face-occupations pairs on decision-making (about 6% in (Duss et al., 2011); about 4% in Ruch et al., 2016). We therefore used a half-normal prior distribution with a mode of 0% (reflecting chance-level performance) and a standard deviation of 5% (expected performance in case of awareness) to calculate BFs (Dienes, 2008, 2014). This yielded a BF of 0.27 for the awareness of faces (mean = 0.17% above chance, standard error of sample SE = 1.27%) and 0.12 for the awareness of occupations (mean = −0.89%, SE = 1.05%). Bayes Factors were below 1/3, suggesting that there is substantial evidence for the null assumption that participants were not aware of masked faces and occupation words. Furthermore, performance in neither of the two tests differed between the condition groups (identical, congruous, incongruous): face awareness test *F*_(2, 84)_ = 0.993, *p* = 0.375, $\eta_{p}^{2}$ = 0.023; occupation awareness test *F*_(2, 84)_ = 0.092, *p* = 0.912, $\eta_{p}^{2}$ = 0.002. Hence, between-group differences discovered in the experiment are unlikely to reflect differences in the awareness of subliminal stimuli.

## Discussion

Decision-making was influenced by both subliminally and supraliminally displayed combinations of faces and written occupations. Income decisions were biased toward the wage category of supraliminally presented occupations, and this bias was either increased or reduced by subliminal encoding, depending on whether subliminal and supraliminal occupations were identical or not. Identical subliminal and supraliminal occupations added up, yielding the strongest observed bias on income decisions, whereas non-identical (incongruous and semantically congruous) occupations canceled each other out, leaving no clearly discernible or even a numerically inverted decision bias (illustrated in Figure 2B). This finding speaks to the power and longevity of subliminally encoded memories because at least 30 min passed between subliminal encoding and decision-making. Post-experimental awareness tests demonstrated that subliminal faces and occupations were presented below the objective threshold of conscious awareness. We deduce from this finding that the subliminal face-occupation combinations presented in the experiment guided decision-making completely outside of conscious awareness. Because participants remembered only about 10% of the supraliminally provided occupation–face combinations, choice behavior in many trials was likely also influenced by supraliminally provided information that was forgotten and hence only unconsciously available. This further emphasizes the role of non-conscious memories in decision-making.

Our results might suggest that subliminally presented faces and occupations were not bound into semantically precise relational memories but merely primed subsequent conscious processing of words and faces due to enhanced perceptual fluency. In fact, the subliminal presentation of faces paired with occupation words instead of consonant strings increased the influence of supraliminal face-occupation pairs on subsequent decisions only if subliminal and supraliminal occupations were identical. Subliminal presentation of non-identical but semantically congruous occupations that reflected the same wage category and a similar field of work (e.g., “historian” and “archeologist”) reduced the supraliminal influence on decisions. This might suggest that subliminal stimulation merely enhanced the perceptual fluency of distinct faces and word forms without leaving a relational memory trace. According to this perspective, an enhanced word and face fluency would facilitate the subsequent conscious encoding and retrieval of identical faces and words which would boost the supraliminal influence on income decisions. Changes in perceptual fluency due to subliminal stimulation might also account for the finding that the supraliminal influence on decision-making was impaired following subliminal presentation of faces paired with non-identical (congruous and incongruous) occupation words compared to consonant strings (control). If meaningful subliminal words undergo more cognitive processing than non-words, words might reduce the resources available for the processing of subliminal faces. Hence, subliminal faces might be better processed in the control than the experimental condition, which would facilitate encoding and retrieval of supraliminal faces and would enhance the supraliminal influence on income decisions. This scenario of perceptual priming thus accounts for both subliminal effects—the enhanced decision bias for identical face-occupation pairs and the reduced decision bias for non-identical pairs. This parsimonious interpretation of results contradicts, however, the well-established fact that one-trial subliminal priming is short-lived and limited to the prime-adjacent target (for a review see Bodner and Masson, 2014). So far, only a few studies reported longer lasting subliminal priming effects. But these could not fully exclude prime awareness (Francken et al., 2011; Muscarella et al., 2013) and they provided only neuronal but no behavioral evidence for priming (Gaillard et al., 2007) or repeatedly presented the same stimulus over many encoding episodes to achieve long-term priming (Chen et al., 2009). Yet, here we observed long-lasting subliminal influences on the behavior in participants who were completely unaware of subliminal stimulation and who received only one subliminal encoding trial. Hence, this pattern of results is atypical for subliminal perceptual face and word priming. Yet, it nicely lines up with previous findings of unconscious relational learning from subliminal stimuli (e.g., Duss et al., 2011; Ruch et al., 2016), as argued below.

Although priming alone might account for our findings, we favor the possibility that subliminally presented face-occupation pairs were actually stored in semantically precise relational memories that guided later decisions. Such a scenario is suggested by previous evidence from experiments with similar designs (Duss et al., 2011; Reber and Henke, 2011; Reber et al., 2012, 2014; Ruch et al., 2016). For example, the subliminal presentation of face-occupation pairs was found to shape subsequent conscious semantic decisions regarding the income, education, and creativity of these same individuals (Duss et al., 2011; Ruch et al., 2016). The subliminal presentation of foreign words with translation words (e.g., “*gumpel* = dog”) also helped participants distinguishing between supraliminally displayed correct and incorrect translations of the same foreign words (e.g., “*gumpel* = hound” vs. “*gumpel* = spear,” see Ruch et al., 2016). As in the present study, only one or two subliminal encoding episodes for each stimulus pair were required to shape decisions that were delayed by up to half an hour. Hence subliminal stimulus pairs were rapidly encoded into lasting memories. Relational encoding of semantic and perceptual associations calls on the hippocampus (Henke et al., 1997; Holdstock et al., 2002; Prince et al., 2005), which is able to rapidly encode information and store relations for long-term. Perceptual priming on the other hand depends on neocortex (Henson, 2003), which requires many identical learning episodes to form lasting memories for single items (McClelland et al., 1995). This explains why subliminal priming studies, most often using single-item stimuli, yielded short-lived subliminal effects (Forster et al., 1990; Ferrand, 1996; Greenwald et al., 1996), whereas studies presenting pairs of stimuli that trigger relational processing yielded lasting subliminal influences. The assumption that long-lasting unconscious associations were formed after a single presentation of a subliminal face-occupation combination contradicts the classic view that consciousness is a precondition for rapid relational learning (Moscovitch, 1995; Squire and Zola, 1996; Tulving, 2002; Mitchell et al., 2009). It is, however, in line with much research implying the contrary (Henke, 2010; Dew and Cabeza, 2011; Hannula and Greene, 2012; Olsen et al., 2012)—in particular that humans can rapidly form and later retrieve unconsciously formed semantic associations (Duss et al., 2011; Ruch et al., 2016) by way of hippocampus (Degonda et al., 2005; Reber et al., 2012; Duss et al., 2014; Züst et al., 2015).

Our assumption that subliminally displayed faces and words yielded semantically precise relational memories raises the question why subliminal stimulation had enhanced the influence of conscious encoding on decision-making in the identical but not the congruous condition. Congruous occupations shared the same wage-category and a similar field of work. However, congruous occupations were not synonymous (e.g., “historian” vs. “archeologist,” or “tailor” vs. “shoemaker”), i.e., they still reflected different professional activities and were associated with different incomes (see Supplementary Table 1 for a list of all occupation words). The semantic distance between congruous occupations was probably too large or too salient for participants to synthesize congruous unconscious and conscious face-occupation memories (e.g., “person X is a tailor” and “person X is a shoemaker”) into an unambiguous mental representation. As a consequence, participants may have failed to integrate the two “congruous” associations to infer a high or a low income.

If unconscious relational learning rather than priming is assumed to account for our results, the question about the mechanism of subliminal influences on decision-making arises. Did subliminally and supraliminally formed memories exert independent effects on decision-making? Did subliminally formed memories modulate the subsequent encoding of supraliminal information? Were subliminally and supraliminally formed memories integrated into one single memory representation or two separate but semantically overlapping memory representations? The current experimental design and the present data cannot distinguish between these mechanisms. To unravel the precise nature of interactions between overlapping unconscious and conscious relational memories, sophisticated neuroimaging techniques in combination with multivariate pattern classifier analyses will be required (Chadwick et al., 2011).

The scope of our findings may be limited by several aspects of the experimental design. First, the main factor of interest—congruency between subliminal and supraliminal information—was varied between rather than within subjects. This opens up for the possibility that individual differences in an unknown variable (e.g., the ability to recognize faces; Wilhelm et al., 2010) rather than congruency between subliminal and supraliminal content accounted for the observed differences between participant groups. However, neither responses in the control condition (which was identical for all participants), nor any of the possible confounding factors that we analyzed varied systematically between participant groups. Hence, there is solid ground to assume that the content of subliminal and supraliminal stimuli rather than a sampling bias accounted for the present results. A further problem is the combination of the implicit income rating task with the explicit memory task within each trial because it might motivate participants to solve the implicit task using explicit memory. This explicit memory-based decision strategy might have prevented participants from responding spontaneously and intuitively, which would have reduced unconscious influences on decision-making. However, only a few participants reported to have employed an explicit memory-based income rating strategy. Finally, our findings are limited by the fact that explicit memory for supraliminally presented information was poor. If the conscious encoding of novel face-occupation associations yielded poor memories, unconscious encoding might have failed, too. Yet, we have repeatedly shown that humans are capable of unconsciously acquiring novel information about subliminally presented face-occupation pairs (Degonda et al., 2005; Duss et al., 2011; Züst et al., 2015; Ruch et al., 2016). Nevertheless, future studies on conscious-unconscious interactions in decision-making may be well-advised to (i) employ within-subjects designs, to (ii) implement independent test-sessions for the assessment of intuitive decision-making and conscious memory retrieval, and to (iii) use stimulus-material that is easier to memorize than novel faces and occupation words.

Our findings confirm the view that unconsciously processed information can be rapidly encoded into long-term memory (Gaillard et al., 2007; Chen et al., 2009; Duss et al., 2011; Chong et al., 2014; Ruch et al., 2016). Ruch et al. (2016) had already reported that subliminally presented faces combined with written occupations were stored in memory for almost half an hour to bias delayed decisions about the income of the depicted persons. Here, subliminally presented face-occupation pairs altered income decisions even if the same faces were later presented supraliminally along with potentially conflicting occupations. This indicates that unconsciously acquired knowledge is robust. Our findings further suggest that decisions are not determined by conscious processes alone but may simultaneously benefit from conscious and unconscious knowledge. While some scholars argue that learning and decision-making are strictly governed by consciousness (e.g., Shanks, 2010; Newell and Shanks, 2014) our data support the view that unconscious processes play a larger role in everyday cognitive functioning than commonly assumed (Rosenthal, 2008; Soon et al., 2008; van Gaal et al., 2012; Hassin, 2013).

## Author contributions

SR and KH developed the study concept and designed the experiment. SR collected and analyzed the data. KH and SR contributed to the final analyses and the interpretation of the data. BH wrote a first draft of the manuscript, KH and SR wrote the final report. All authors approved the final version of the manuscript for submission.

### Conflict of interest statement

The authors declare that the research was conducted in the absence of any commercial or financial relationships that could be construed as a potential conflict of interest. The reviewer GH and handling Editor declared their shared affiliation.

## Supplementary material

Supplementary Video 1 Video of one subliminal encoding trial. Video must be played at 60 fps to provide an accurate representation of the stimulus sequence. The embedded subliminal image consists of a male face and the written word “Manager.” A schematized image of a face instead of one of the original faces is used. The individual depicted in this video gave written informed consent to publication of his image.

Supplementary Video 2 Video highlighting the subliminal image in one subliminal encoding trial. This video is identical to Supplementary Video 1, but the contrast of stimulus masks was reduced to enhance the visibility of the subliminal images. These masks were not used in our experiment. The individual depicted in this video gave written informed consent to publication of his image.

Supplementary Table 1 List of all occupation words used in the current study. Word-frequency obtained from the Leipzig Corpora Collection (http://corpora.uni-leipzig.de/), word length (number of letters), and mean estimated income as rated by an independent sample of 34 participants are indicated. Words are grouped by stimulus lists (A, A′, B, and B′) that were used to counterbalance stimuli across conditions, and by wage-category. Pairs of semantically congruous occupation words are labeled with the same identification number.

Supplementary Table 2 List of all consonant strings with corresponding word lengths (number of letters).
