# Supplementary material for: Subliminally and Supraliminally Acquired Long-Term Memories Jointly Bias Delayed Decisions
Source: Front Psychol. 2017 Sep 12;8:1542. doi: 10.3389/fpsyg.2017.01542 (PMC5600932; doi:10.3389/fpsyg.2017.01542)
Supplement: Supplementary Table 1 — List of all occupation words used in the current study. Word-frequency obtained from the Leipzig Corpora Collection (http://corpora.uni-leipzig.de/), word length (number of letters), and mean estimated income as rated by an independent sample of 34 participants are indicated. Words are grouped by stimulus lists (A, A′, B, and B′) that were used to counterbalance stimuli across conditions, and by wage-category. Pairs of semantically congruous occupation words are labeled with the same identification number. [file Table1.PDF]

Supplementary Table 1

Occupation	Pair_ID	Stimulus_List	Wage_Category	W_Frequency	NLetters	Income_MEAN*	Income_SD*
Hotelier	1	A	high	294	8	0.5995	0.5315
Gastronom	1	A'	high	243	9	0.1791	0.5495
Archäologe	2	A	high	238	10	0.3976	0.5614
Historiker	2	A'	high	2428	10	0.4067	0.4240
Übersetzer	3	A	high	534	10	0.4192	0.5391
Dolmetscher	3	A'	high	520	11	0.6243	0.5887
Pharmazeut	4	A	high	24	10	0.6155	0.6183
Apotheker	4	A'	high	982	9	0.5807	0.6138
Informatiker	5	A	high	378	12	0.6308	0.5959
Programmierer	5	A'	high	387	13	0.6196	0.6468
Architekt	6	A	high	2292	9	1.0609	0.6100
Ingenieur	6	A'	high	1517	9	1.0169	0.5351
Professor	7	A	high	7877	9	1.3464	0.4463
Dozent	7	A'	high	339	6	0.8265	0.6826
Finanzberater	8	A	high	234	13	0.8694	0.5177
Bankier	8	A'	high	195	7	1.3065	0.4351
Diplomat	9	A	high	1327	8	1.3019	0.4788
Botschafter	9	A'	high	4354	11	1.3637	0.4879
Richter	10	A	high	17274	7	1.5150	0.3788
Anwalt	10	A'	high	8271	6	1.5988	0.3406
Knecht	11	A	low	199	6	-1.5840	0.3970
Diener	11	A'	low	487	6	-1.1208	0.5222
Müllmann	12	A	low	46	8	-1.2740	0.5238
Strassenreiniger	12	A'	low	23	16	-1.3368	0.3838
Lagerarbeiter	13	A	low	45	13	-1.2903	0.4346
Möbelpacker	13	A'	low	32	11	-1.2488	0.4813
Kassierer	14	A	low	163	9	-1.1032	0.5173
Verkäufer	14	A'	low	1863	9	-1.0214	0.4679
Briefträger	15	A	low	147	11	-0.7955	0.4673
Kurier	15	A'	low	520	6	-1.0935	0.5510
Gärtner	16	A	low	929	7	-0.7434	0.4151
Florist	16	A'	low	20	7	-0.9460	0.4987
Maurer	17	A	low	1389	6	-0.5999	0.6104
Maler	17	A'	low	2485	5	-0.7245	0.4937
Landwirt	18	A	low	952	8	-0.7193	0.7009
Bauer	18	A'	low	3176	5	-0.7845	0.5560
Wärter	19	A	low	234	6	-0.8468	0.5596
Aufseher	19	A'	low	286	8	-0.4230	0.5947
Fleischer	20	A	low	409	9	-0.4865	0.4152
Metzger	20	A'	low	716	7	-0.6163	0.4584
Geologe	21	B	high	136	7	0.3610	0.5349
Geograph	21	B'	high	39	8	0.4141	0.6549
Moderator	22	B	high	2412	9	0.5776	0.5130
Journalist	22	B'	high	2705	10	0.4153	0.4599
Beamter	23	B	high	1492	7	0.4981	0.7437
Staatsdiener	23	B'	high	213	12	0.4134	0.4732
Mathematiker	24	B	high	464	12	0.6589	0.5003
Statistiker	24	B'	high	1041	11	0.5285	0.6631
Astronom	25	B	high	126	8	0.4300	0.7679
Physiker	25	B'	high	1253	8	0.8967	0.4436

Supplementary Table 1

Produzent	26	B	high	1662	9	1.0122	0.4548
Regisseur	26	B'	high	8801	9	0.7141	0.5719
Unternehmer	27	B	high	6152	11	1.0611	0.4817
Investor	27	B'	high	3829	8	1.1155	0.6096
Direktor	28	B	high	6221	8	1.3811	0.4975
Manager	28	B'	high	16632	7	1.3669	0.3985
Doktor	29	B	high	944	6	1.4640	0.4090
Chirurg	29	B'	high	334	7	1.4281	0.5926
Kanzler	30	B	high	13001	7	1.4788	0.4870
Bundesrat	30	B'	high	5585	9	1.5077	0.4805
Tellerwäscher	31	B	low	50	13	-1.5389	0.3835
Küchenhilfe	31	B'	low	31	11	-1.6115	0.3426
Hirte	32	B	low	109	5	-1.4983	0.4129
Schäfer	32	B'	low	2724	7	-1.3643	0.4139
Buschauffeur	33	B	low	948	12	-0.8330	0.3914
Taxifahrer	33	B'	low	1227	10	-1.0745	0.3918
Hausmeister	34	B	low	668	11	-0.8933	0.5695
Platzwart	34	B'	low	86	9	-0.9875	0.6859
Coiffeur	35	B	low	16	8	-1.0321	0.5858
Kosmetiker	35	B'	low	2	10	-0.7142	0.5854
Schneider	36	B	low	6177	9	-0.7157	0.4966
Schuster	36	B'	low	2117	8	-0.9612	0.5005
Jäger	37	B	low	2548	5	-0.9137	0.5586
Förster	37	B'	low	945	7	-0.4321	0.4521
Schweisser	38	B'	low	72	10	-0.6627	0.4058
Schmied	37	B	low	230	7	-0.6439	0.4391
Zimmermann	39	B	low	1808	10	-0.5176	0.4525
Schreiner	39	B'	low	791	9	-0.6436	0.4249
Konditor	40	B	low	61	8	-0.4792	0.5673
Bäcker	40	B'	low	721	6	-0.5970	0.4432

**A total of 34 participants indicated for each occupation word whether a male person with the given occupation would earn a low or a high income. Income ratings were obtained using a scale ranging from -2 (low income) to +2 (high income).*
