# Supplementary material for: Subliminally and Supraliminally Acquired Long-Term Memories Jointly Bias Delayed Decisions
Source: Front Psychol. 2017 Sep 12;8:1542. doi: 10.3389/fpsyg.2017.01542 (PMC5600932; doi:10.3389/fpsyg.2017.01542)
Supplement: Supplementary Table 2 — List of all consonant strings with corresponding word lengths (number of letters). [file Table2.PDF]

Supplementary Table 2

Consonant_Strings	NLetters
Sxrhrfr	7
Brrtrcgr	8
Pzndrhs	7
Rcbrhd	6
Knhrmttmh	9
Tsttmgrslmtpf	13
Pmrnsntg	8
Grrrwrdr	8
Bnrksktstrgh	12
Rrnttrvmnk	10
Mthsrlrst	9
Mftrrrkmsl	10
Llrlcsrrbtn	11
Hsclrrzntcp	11
Cfrlnrthg	9
Drnrrshrwt	10
Fntncttr	8
Dcrckft	7
Khrsfh	6
Szgkrrnrs	9
